# A supervised learning regression method for the analysis of oral sensitivity of healthy individuals and patients with chemosensory loss

**DOI:** 10.1038/s41598-023-44817-w

**Published:** 2023-10-16

**Authors:** Lala Chaimae Naciri, Mariano Mastinu, Melania Melis, Tomer Green, Anne Wolf, Thomas Hummel, Iole Tomassini Barbarossa

**Affiliations:** 1 Department of Biomedical Sciences, University of Cagliari, Monserrato, CA Italy; 2 Smell & Taste Clinic, Department of Otorhinolaryngology, TU Dresden, Dresden, Germany; 3 Institute of Biochemistry, Food Science and Nutrition, The Hebrew University of Jerusalem, Rehovot, Israel

**Keywords:** Computational biology and bioinformatics, Neuroscience, Health care

## Abstract

The gustatory, olfactory, and trigeminal systems are anatomically separated. However, they interact cognitively to give rise to oral perception, which can significantly affect health and quality of life. We built a Supervised Learning (SL) regression model that, exploiting participants’ features, was capable of automatically analyzing with high precision the self-ratings of oral sensitivity of healthy participants and patients with chemosensory loss, determining the contribution of its components: gustatory, olfactory, and trigeminal. CatBoost regressor provided predicted values of the self-rated oral sensitivity close to experimental values. Patients showed lower predicted values of oral sensitivity, lower scores for measured taste, spiciness, astringency, and smell sensitivity, higher BMI, and lower levels of well-being. CatBoost regressor defined the impact of the single components of oral perception in the two groups. The trigeminal component was the most significant, though astringency and spiciness provided similar contributions in controls, while astringency was most important in patients. Taste was more important in controls while smell was the least important in both groups. Identifying the significance of the oral perception components and the differences between the two groups provide important information to allow for more targeted examinations supporting both patients and healthcare professionals in clinical practice.

## Introduction

Oral perception is commonly used as the term to define the combinations of the multisensory interactions of taste, smell, and the trigeminal system that we perceive when tasting food. Smell and taste also significantly impact how people interact with their environment, altering their behaviors^1^. While gustatory, olfactory, and trigeminal systems are anatomically separated with different functions, this does not necessarily mean that they cannot interact on a cognitive level^2^. As a consequence, people frequently report a loss of their sense of taste when their nose is blocked, which is only one common example illustrating that the senses of taste and smell are frequently confused.

Individual differences in oral sensitivity are significant, and they can substantially impact dietary preferences and nutritional status^3^. In addition, numerous diseases and medical interventions can have an impact on oral sensitivity^4–9^. This may have significant effects on the quality of life^10,11^. Thus, in clinical practice, assessments of chemosensory function are crucial because they define the quality and quantity of gustatory loss allowing for their separation from possible olfactory dysfunction.

Available clinical tests for taste function rely on the subjectivity of participants since they ask them to identify a taste quality or rate their intensity. In addition, significant time is required to provide reliable taste measures, especially when the assessment is extended to different parts of the tongue (anterior and posterior; left and right) to cover the lateralized sensory innervation of the tongue by different nerves. Since people might have a reduced sensation for specific taste qualities, also called hypogeusia, common approaches require the analysis of all taste stimuli (sweet, sour, bitter, salty, umami) in ascendant concentrations. These techniques are time-consuming and, if based on liquid taste stimuli, require the preparation of the required solutions the day before because of their short shelf life.

Another approach is based on the evaluation of taste performance by asking participants to rate their sense of taste. Subjective assessments of taste function are crucial to determine the consequence of impairment in patients’ daily life. However, the self-rated results are controversial due to the general lack of knowledge of taste sensation per se which, as above said, often being confused with decreased flavor perception. Patients reporting a taste dysfunction often have a deficit in olfactory perception from decreased retronasal olfactory stimulation^12^, which does not allow them to perceive the ‘flavor’ of coffee, for example. Data reported in the literature show that patients might not respond correctly to these types of questions^13^, also given the low consciousness of taste problems in those that have reduced taste function^7,12^.

In the case of self-assessment procedures, questions should be detailed to detect more information also regarding olfaction, and trigeminal sensation. Numerous studies reported a direct influence of trigeminal activation, e.g., by capsaicin, on oral perception^14–16^. Together with the evaluation of the astringency sensation of dryness, for example, evoked by tannins in foods^17^, the perception of spiciness gives a measure of the general trigeminal function^18^. On the other hand, factors like lifestyle, profession, age, gender, and pre-existing illness might influence awareness of oral perception^13^. Unlike olfactory tests^19^, standardized and reliable taste tests are available, but they are not used. This may be because these tests are too complex, and possibly time-consuming for the caregivers. Hence it appears logical that new procedures should be examined.

Recently, we used a Supervised Learning (SL) regressor model as a reliable approach to thoroughly study the taste function of healthy individuals and patients with chemosensory loss and characterize the combination of their responses that best predicted the overall taste statuses in the two groups^20^. Given its importance in nutrition, considering that the oral perception that we experience when tasting food consists of the multimodal interactions of taste, smell, and the trigeminal system, here we assessed the efficacy of SL regression methods to analyze with high precision the subjective ratings of the oral sensitivity of healthy participants and patients with chemosensory loss. The proposed approach was also used to determine the significance and contribution to the oral sensitivity of the single components of taste, olfactory, and trigeminal, also highlighting the differences between the two groups. Besides, the impact on the oral sensitivity of the taste quality and quantity and that of other parameters that may influence oral sensitivity were also evaluated.

## Material and methods

### Participants

One-hundred fifty-two individuals aged 18 to 81 years (38.3 ± 14.3 years; 103 females) have been invited to participate in the study. They were recruited at the Department of Otorhinolaryngology of the TU Dresden. A chemosensory impairment was self-reported by 50 participants who were Smell and Taste Clinic patients. One hundred and two healthy individuals were included in the study's sample as a control group if they self-reported a good sense of taste. They were recruited from the TU Dresden community and matched by age. In all subjects a structured history was taken. The study size was determined by the statistical power analysis using the software G*Power, Dusseldorf, Germany. Pregnancy, an allergy to study-related substances, untreated hypo- or hyperthyreosis, uncontrolled diabetic mellitus, kidney failure, and serious cardiovascular conditions were among the exclusion criteria. Prior to their enrollment in the study, all participants gave their informed, written consent. The research protocol was approved by the Ethics Review Board at the University Clinic of the Technische Universität Dresden, application number BO-EK-25012021, and it was performed in accordance with the Declaration of Helsinki.

### Study design

**Figure 1:**
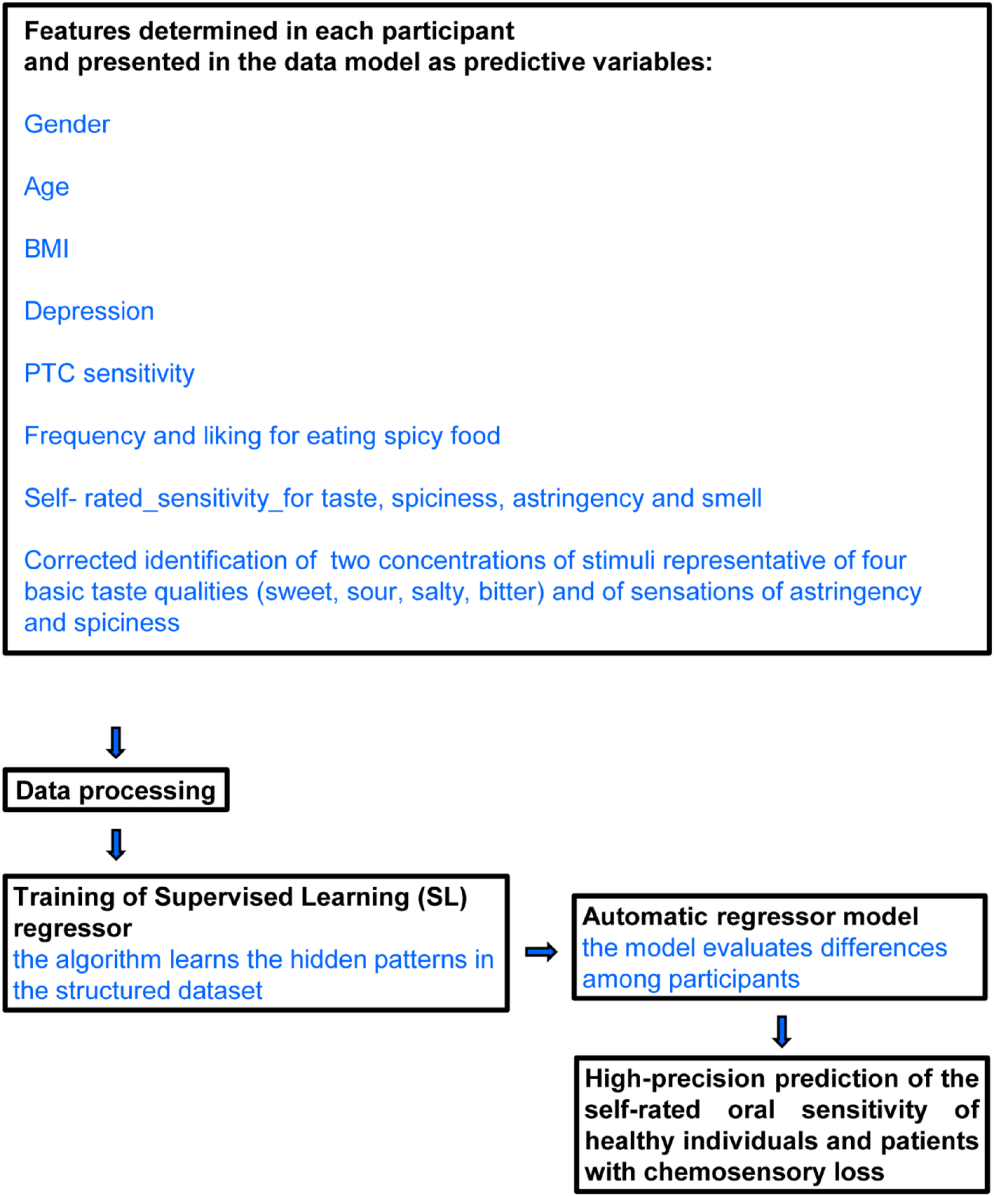
Graphic diagram representing the study design.

The experimental value of self-rated oral sensitivity was calculated in each participant as the mean value of self-rated taste sensitivity, self-rated sensitivity for spiciness, and self-rated sensitivity for astringency. The prediction values of the self-rated oral sensitivity of control individuals and patients with chemosensory loss were performed by the SL regressors. The SL regressors learn from data (presented in the model as predictive variables), create automatic regressor models that evaluate the differences among participants, and return a high-precision prediction of the target in the participants of the two groups. To this aim, the dataset was randomly divided into training data (80%) and test data (20%). During training, the algorithm searches for patterns that correlate with the target in the two groups, then it takes new unknown inputs (in the test data) to determine the predictive values of the target in the two groups. The following algorithms were used: Logistic Regression, Random Forest Regressor, and CatBoost Regressor. Two SL experiments were performed: the first included control individuals (n = 102), and the second included patients with chemosensory loss (n = 50). The data presented in the model as predictive variables were the biological features that were previously recorded from each participant as described below. Figure 1 shows the study design of each SL experiment.

Data processing operations and the resolution of the overfitting or underfitting problems, which are crucial phases in the running of an SL experiment, were performed according to Naciri et al.^21,22^. They included handling of missing values; elimination of the duplicate values; the converting of the dataset's content into a structure that machine learning algorithms can employ; the models’ hyperparameter optimization that allows finding the optimal value of the number of model iterations, learning rate, and depth of the SL regressors.

Symmetric Mean Absolute Percentage Error (SMAPE) and Mean Squared Error (MSE), which denote the differences between the observed and predicted values, were used to evaluate the performance of the algorithms. Specifically, SMAPE assesses the error percentage of predicted values and MSE assesses the average of the sum of the squared difference between the observed and the predicted values.

The interpretation of the output of the best SL model (CatBoost regressor) was evaluated by the Permutation explainer method which is a default model agnostic explainer used for structured datasets, that has important performance optimizations and does not require regularization parameter tuning. The Permutation explainer works by iterating over complete permutations of the features forward and reversed. The permutation of one feature at a time ensures the efficiency of the original model that we choose to use for the approximation of the contribution of a feature value. The Permutation explainer can be used for Local Interpretability and Global Interpretability^23,24^:

- In the case of Local Interpretability, which regards a single instance, the Permutation explainer returns the plot of the single instance representing the importance and contribution of features in the CatBoost regressor model learning to predict the target value. In the plot, the average of the predicted values of the dataset (E[f(x)]) is the starting point to determine the contribution of each feature.
- In the case of Global Interpretability, which regards the whole dataset, the Permutation explainer returns the global summary plot representing the average of the importance and the contribution (mean of SHAP values) of features in the learning of the CatBoost regressor model to predict the values of the target.

### Experimental procedure for the biological feature measurements

The experimental procedure for the biological feature measurements was completed in a single session. For at least half an hour prior to testing, participants were instructed to refrain from eating, drinking anything save water, and using chewing gum or other dental care items. Participants' health status was evaluated before the session using a thorough medical history. Participants were asked to self-report their weight (kg) and height (m) to determine their BMI (kg/m^2^). All participants self-rated their sensitivity for taste, spiciness, astringency, and smell using a 7-point Likert-type scale and they were requested to indicate their frequency and liking for eating spicy food. Moreover, participants were tested for their depression status by using the 5-item World Health Organization Well-Being Index (WHO-5)^25,26^. Each item response was rated on a 6-point Likert scale from 0 to 5, based on the severity, giving scores ranging from 0 to 25. A high score denotes a high level of well-being, whereas a score of less than 13 denotes a low level of well-being^25,26^.

### Sensory measurements

All measurements of oral sensitivity were performed by a modification of the “Taste Strip Test” (TST, Burghart Company, Holm, Germany)^27,28^. Briefly, the filter papers were impregnated with two concentrations (one low and one high) of stimuli representative of four basic taste qualities (sweet, sour, salty, bitter) and of stimulants capable of evoking sensations of astringency and spiciness. The two concentrations for each stimulus were: 0.4 and 0.05 g/mL sucrose; 0.3 and 0.05 g/mL citric acid; 0.25 and 0.016 g/mL sodium chloride; 0.006 and 0.0004 g/mL quinine hydrochloride; 0.1 and 0.2 g/mL tannin; 2.47 × 10^–5^ and 2.47 × 10^–4^ g/mL capsaicin. After placing each filter paper on the tongue, each participant had to identify the taste quality of each stimulus by choosing from a list of six possible choices (sweet, sour, salty, bitter, astringent and hot) in a six-alternative forced-choice procedure (compare to^27^). Taste stimuli were presented in a semi-randomized manner. However, trigeminal stimuli were presented as last due to their persistent effect. Participants were instructed to rinse their mouths with tap water before each new test. For each participant, the entire process took 20 min.

Each correct answer was rated 1, thus the maximum score given when all stimuli were correctly identified was 12. The features that represent the score given for each stimulus are called from now on salty_low_taste_correct; salty_high_taste_correct; sweet_low_taste_correct; sweet_high_taste_correct; sour_low_taste_correct; sour_high_taste_correct; bitter_low_taste_correct; bitter_high_taste_correct; hot_low_taste_correct; hot_high_taste_correct; astring_low_taste_correct; astring_high_taste_correct. Whereas the feature that represents the sum of scores given for all stimuli is called score_lowhigh_capsadstr1.

PTC-impregnated filter paper (phenylthiocarbamide test paper; Sensonics, Philadelphia, PA, USA)^29^ was used to assess the bitter taste of PTC, whose taste sensitivity is highly correlated to the bitter perception of 6-n-propylthyouracil (PROP)^30,31^. In order to re-focus the participant’s attention on the experimental procedures, the intensity of the PTC-related bitterness was rated on a different scale than the other tastes, ranging from 0 to 10 (where 10 denoted the most intense bitterness).

### Statistical analysis

Fisher’s Exact Test was used to analyze differences, between control individuals and patients with chemosensory loss, in the frequency of correct answers for the two concentrations of each stimulus, gender and liking for eating spicy food (liketoeatspicy). Data were checked for normality with Shapiro–Wilk test and Kolmogorov–Smirnov test. Since the normality was violated the Mann–Whitney U Test was used to compare differences, between the two groups, in age, BMI, depression, total taste score (score_lowhigh_capsadstr1), self-rated taste sensitivity (selfratedsensitivitytaste), self-rated sensitivity for spiciness (selfratedsensitivityspicyness), self-rated sensitivity for astringency (selfratedsensitivityastringency), self-rated smell sensitivity (selfratedsensitivitysmell) and frequency for eating spicy food (spicyfoodmealspermonth), and the relationships between the self-rated oral sensitivity data and oral sensitivity data obtained by “Taste Strip Test” (score_lowhigh_capsadstr1) in the two groups were assessed using Spearman’s correlation analysis. Statistical analyses were conducted using STATISTICA for WINDOWS (version 7; StatSoft Inc, Tulsa, OK, USA). P values < 0.05 were considered significant.

## Results

Table 1 shows the differences in mean values ± SE or the number of correct answers or participants’ frequency of biological features between control individuals and patients with chemosensory loss. Mann–Whitney U Test showed that the total taste score (score_lowhigh_capsadstr1), as well as the self-rated sensitivity for taste, spiciness, astringency and smell were higher in control individuals than in patients with chemosensory loss (p < 0.0001). Instead, the BMI and depression status were higher in patients than in controls (p ≤ 0.035). The number of correct answers for the low concentrations of salty, bitter, hot and astringency and the high concentration of sweet, sour and astringency, as well as the number of participants who like to eat spicy, were higher in controls than in patients (χ^2^ ≥ 4.94; p ≤ 0.039; Fisher’s test). No differences in the low concentrations of sweet and sour, and high concentrations of salty, bitter and hot or in PTC ratings, frequency of eating spicy food, age and gender were found between the two groups (p > 0.05).

**Table 1:** Differences in biological features between control individuals and patients with chemosensory loss.

Features	Controls (*n* = 102)	Patients (n = 50)	p-value
score_lowhigh_capsadstr1	9.37 ± 0.21	7.14 ± 0.30	< 0.001
PTC1	5.42 ± 0.36	4.55 ± 0.52	0.146
Age (years)	36.70 ± 1.38	40.34 ± 1.97	0.074
BMI (kg/m^2^)	23.66 ± 0.46	25.60 ± 0.65	0.035
depression	17.15 ± 0.42	14.20 ± 0.60	< 0.001
Self-rated sensitivity taste	3.79 ± 0.11	1.90 ± 0.15	< 0.001
Self-rated sensitivity spiciness	4.09 ± 0.11	2.80 ± 0.16	< 0.001
Self-rated sensitivity astringency	3.77 ± 0.11	2.64 ± 0.16	< 0.001
Self-rated sensitivity smell	3.62 ± 0.12	1.90 ± 0.15	< 0.001
Spicy food meals per month	4.92 ± 0.59	1.70 ± 0.17	0.379
salty_low_taste_correct/non (n)	87/15	32/18	0.003
salty_high_taste_correct/non (n)	90/12	44/6	0.579
sweet_low_taste_correct/non (n)	72/30	31/19	0.189
sweet_high_taste_correct/non (n)	100/2	45/5	0.039
sour_low_taste_correct/non (n)	32/70	9/41	0.058
sour_high_taste_correct/non (n)	93/9	35/15	0.001
bitter_low_taste_correct/non (n)	63/39	21/29	0.017
bitter_high_taste_correct/non (n)	77/25	38/12	0.557
hot_low_taste_correct/non (n)	77/25	17/33	0.000
hot_high_taste_correct/non (n)	97/5	46/4	0.346
astring_low_taste_correct/non (n)	78/24	15/35	< 0.001
astring_high_taste_correct/non (n)	86/16	24/26	< 0.001
Male/Female *(n)*	37/65	14/36	0.203
Liketoeatspicy/non (*n*)	66/36	22/28	0.010

Values are means ± SE, or numbers of correct answers, or of participants. Significant differences in mean values were determined by Mann–Whitney U Test (p ≤ 0.035), while significant differences in the number of correct answers or participants’ frequency were determined by Fisher's method (*p* < 0.039).

**Figure 2:**
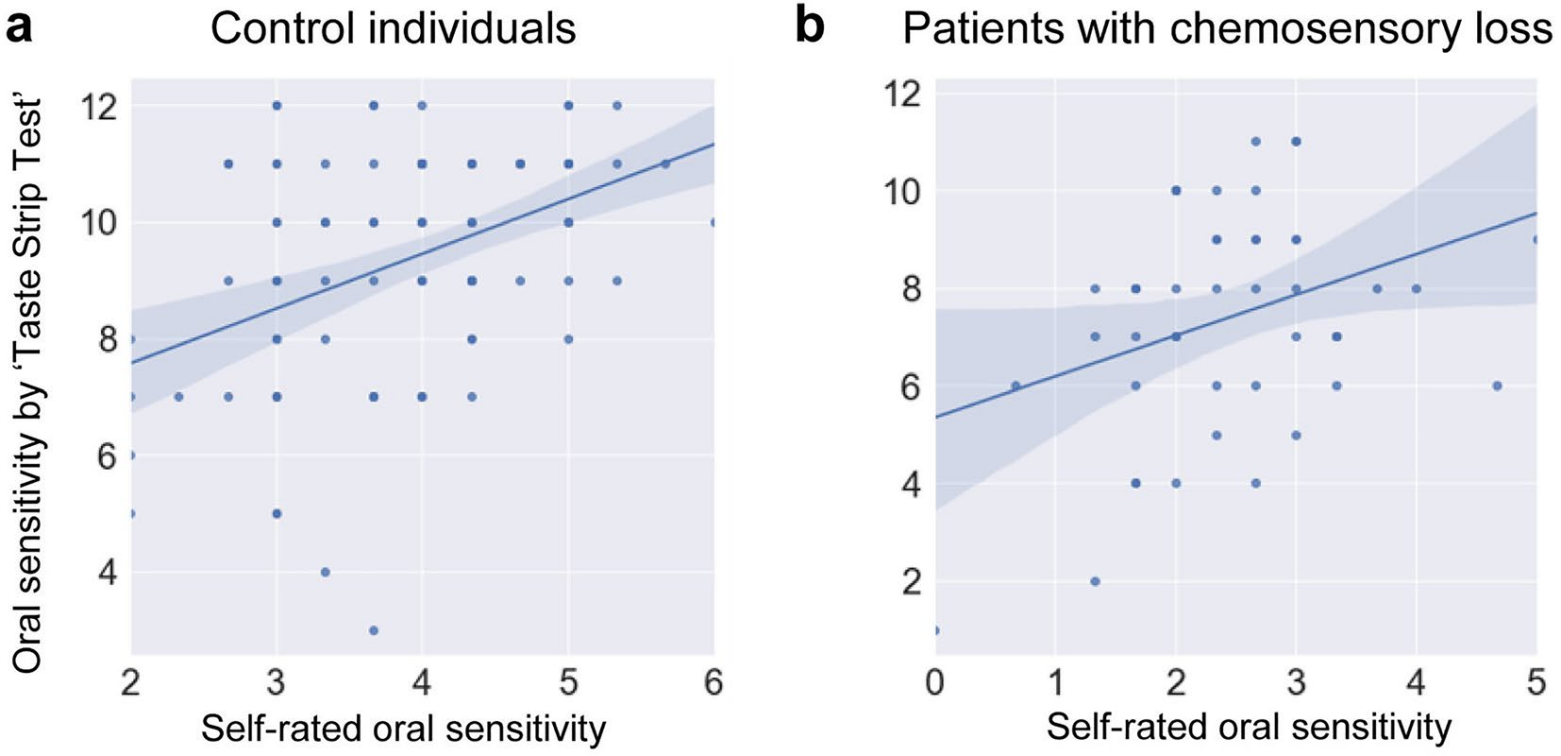
Relationships between the self-rated oral sensitivity data and oral sensitivity data obtained by the “Taste Strip Test” (score_lowhigh_capsadstr1) in the control individuals (n = 102) (**a**) and in patients with chemosensory loss (n = 50) (**b**).

Figure 2 shows the scatterplots depicting the relationships between the self-rated oral sensitivity data and oral sensitivity data obtained by the “Taste Strip Test” (score_lowhigh_capsadstr1) in the control individuals (**a**) and in patients with chemosensory loss (**b**). Spearman’s correlation analysis showed that the self-rated oral sensitivity data were correlated with the oral sensitivity data obtained by the “12 Taste Strip Test” (r ≥ 0.34; p ≤ 0.017) in the two groups. No correlation was found between self-rated oral sensitivity data and PTC bitterness (p > 0.05).

**Figure 3:**
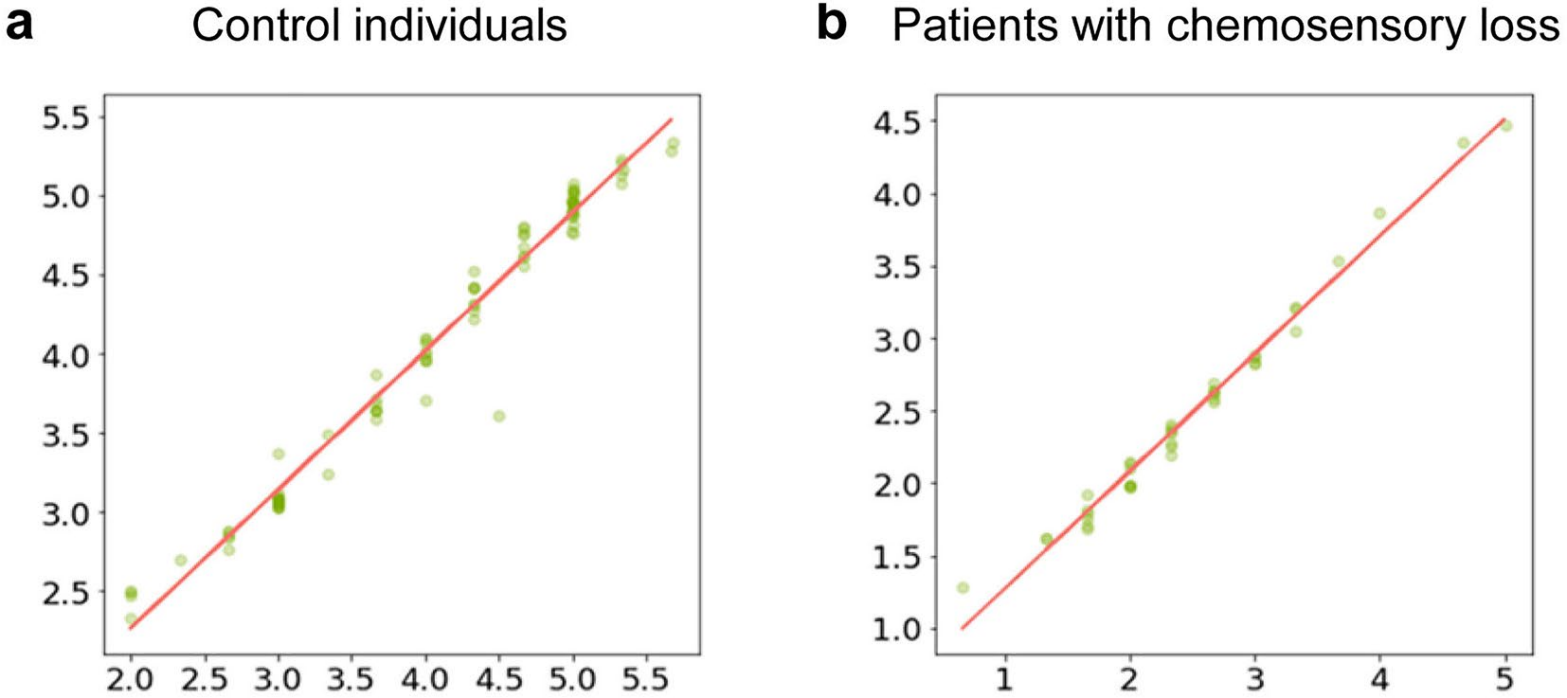
Scatterplots of the self-rated oral sensitivity experimental values vs. predicted values obtained with the CatBoost regressor in the control individuals (n = 102) (**a**) and in patients with chemosensory loss (n = 50) (**b**).

The metrics of evaluation of the training and performance of the algorithms, SMAPE and MSE, showed that the CatBoost regressor algorithm was the best algorithm to predict the values of the self-rated oral sensitivity with high precision. The scatterplots in Fig. 3 show experimental values vs. predicted values of the self-rated oral sensitivity obtained with the CatBoost regressor in controls (a) and patients with chemosensory loss (b). The values of the MSE, which assesses the average squared difference between the experimental and predicted values, were 0.025 and 0.034 in controls and patients, respectively. The values of the SMAPE, which represent the error percentage of predicted values, were 5.39%, and 27.81% in control individuals and patients with chemosensory loss, respectively.

**Figure 4:**
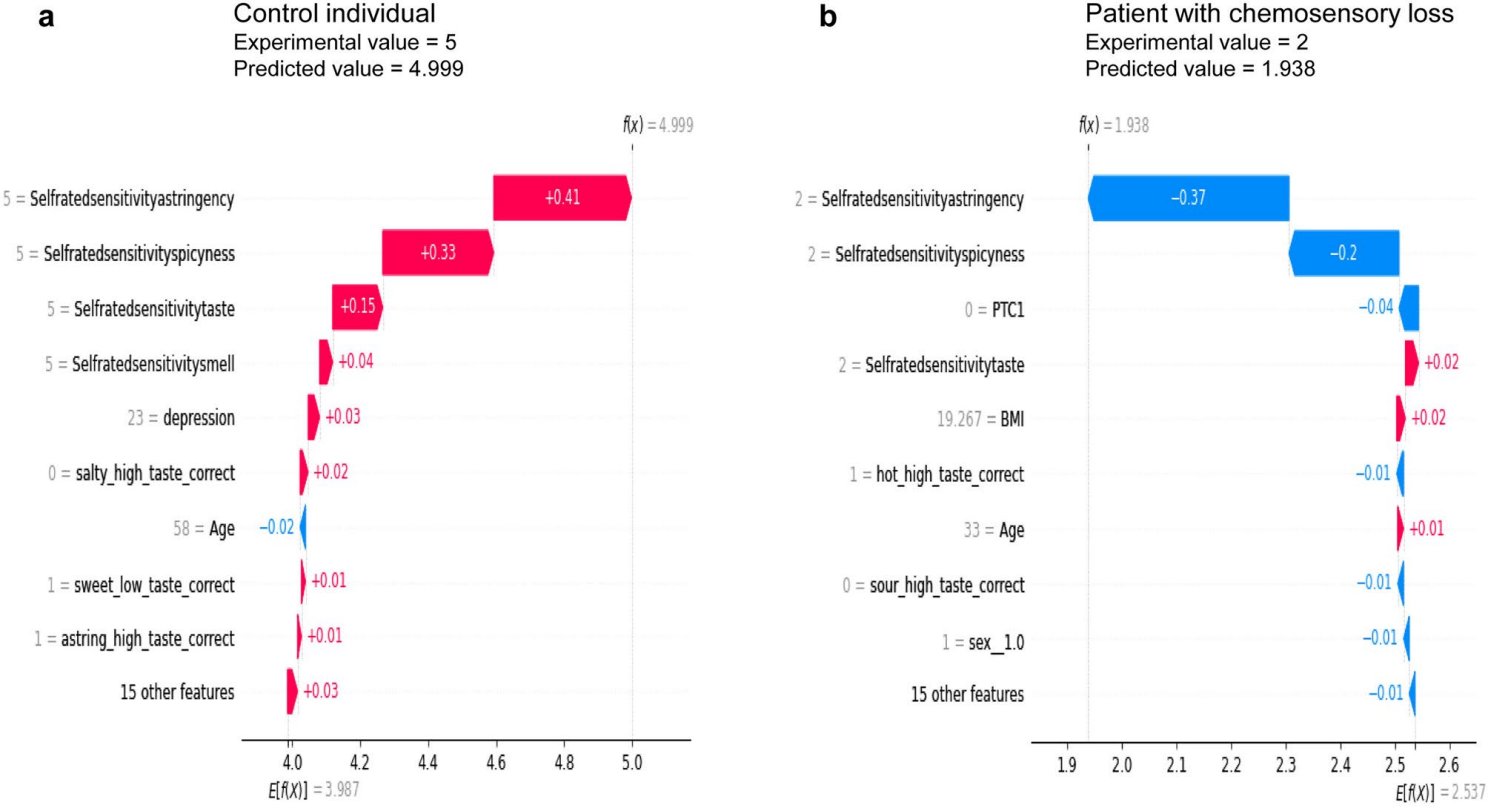
Plots of single instances representing the importance and contribution of features in the learning of the CatBoost regressor model to predict the value of the self-rated oral sensitivity in a control individual (**a**) and in a patient with chemosensory loss (**b**). The Y axis represents the order of the features’ importance, while the X axis represents the contribution of features. Below the X axis, (E[f(x)]) is the average of the predicted values of the dataset which is the starting point to determine the contribution of each feature. At the top of the plot, f(x) is the predicted value in the single instance. The numbers next to each bar represent each feature’s contribution values.

Figure 4 shows the plots of single instances representing the importance and contribution of features in the CatBoost regressor model learning to predict the self-rated oral sensitivity value in a representative control individual (a), in which the experimental value was 5 and the predicted value was 4.999, and in a representative patient (b), in which the experimental value was 2 and the predicted value was 1.938. The average of the predicted values in the control group (E[f(x)]) was higher (3.987) than that of the patient group (2.537). In addition, in the control individual, the self-rated sensitivity for astringency (selfratedsensitivityastringency), spiciness (selfratedsensitivityspicyness), taste (selfratedsensitivitytaste) and smell (selfratedsensitivitysmell) were the four important features and gave a contribution of + 0.41, + 0.33, + 0.15 and + 0.04. The successive five features in importance order were: depression, salty high_taste_correct, age, sweet_low_taste correct, and astringency_high_taste correct. Differently, in the patient, the self-rated sensitivity for astringency (selfratedsensitivityastringency), spiciness (selfratedsensitivityspicyness), PTC, and taste (selfratedsensitivitytaste) were the four important features and gave a contribution of − 0.37, − 0.2, − 0.04 and + 0.02. The successive five features in importance order were: BMI, hot_high_taste correct, age, sour high_taste_correct, and sex.

**Figure 5:**
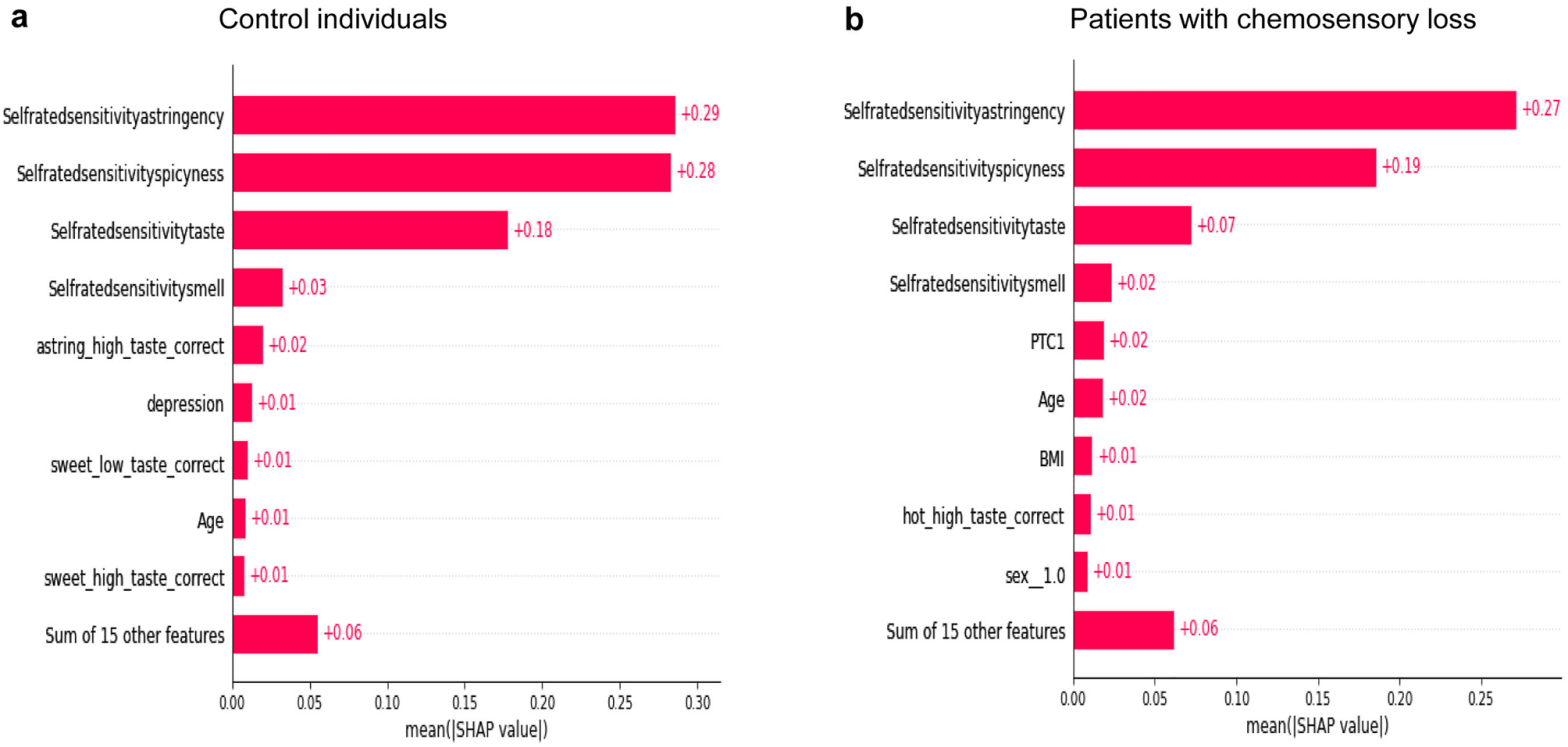
Global summary plots representing the average of the importance and the contribution of features in the learning of the CatBoost regressor model to predict the values of the self-rated oral sensitivity in control individuals (**a**) and in patients with chemosensory loss (**b**). The Y axis represents the order of the features’ importance, while the average contribution to the model output is represented in the X axis. The numbers next to each bar represent the mean values of the contribution of features.

Figure 5 shows the global summary plots representing the average of the importance and contribution (mean of SHAP values) of features in the learning of the CatBoost regressor model to predict the values of the self-rated oral sensitivity in control individuals (a) and in patients with chemosensory loss (b). The self-rated sensitivity for astringency (selfratedsensitivityastringency) was the most important feature and gave a similar contribution in the two groups (+ 0.29 and + 0.27). The self-rated sensitivity for spiciness (selfratedsensitivityspicyness) was the second important feature in both groups, but contributed more in facilitating the learning of the model in control individuals (+ 0.28) than in patients (+ 0.19). The self-rated taste sensitivity (selfratedsensitivitytaste) was the third important feature in the two groups and contributed more in control individuals (+ 0.18) than in patients (+ 0.07). The self-rated smell sensitivity (selfratedsensitivitysmell) was the fourth feature in both groups giving a similar contribution in the learning of the model (+ 0.03 and + 0.02). The successive five features in order of importance were different in the two groups and were: astring_high_taste_correct, depression, sweet_low_taste correct, age and sweet_high_taste correct in control individuals and PTC1, age, BMI, Hot_high_taste correct and sex in patients with chemosensory loss.

## Discussion

The primary aim of the present work was to build an SL model capable of automatically analyzing with high precision the subjective ratings of the oral sensitivity of healthy individuals and patients with chemosensory loss considering its three components: gustatory, olfactory, and trigeminal functions. To this aim, the prediction values of the self-rated oral sensitivity of control individuals and patients with chemosensory loss were obtained by the Supervised Learning (SL) Regressions. The following SL Regression algorithms (Logistic Regression, Random Forest Regressor, and CatBoost Regressor) were used as they are specifically designed to forecast continuous outcomes like self-rated oral sensitivity^32^. In the proposed approach, the algorithms learn from data that were participants’ biological features previously determined (presented in the model as predictive variables), create automatic regressor models that evaluate the differences among participants, and return a high-precision prediction of the self-rated oral sensitivity in the participants of the two groups. The self-rated oral sensitivity that we used as the target was calculated in each participant as the mean value of the self-rated taste sensitivity, self-rated sensitivity for spiciness, and self-rated sensitivity for astringency. The approach allowed us to deeply understand the importance and contribution of the taste, olfactory, and trigeminal components of oral sensitivity, highlighting the differences between the two groups. In addition, the method enabled us also to determine the importance and impact on the model output of the six stimuli presented to each participant, as well as of their concentration, and other parameters that may influence oral sensitivity. To achieve the goal of this work, we performed two SL experiments, one included control individuals and the other included participants who were selected based on their self-reported chemosensory impairments. The lower values of the total taste, the self-rated taste sensitivity, the self-rated sensitivity for spiciness, the self-rated sensitivity for astringency, and the self-rated smell sensitivity, as well as the average of the predicted values (E[f(x)]) in these participants, compared to those of participants of the control group, confirmed that they had chemosensory impairments. These participants showed a higher BMI. This was expected because reductions in taste sensitivity for sweet, umami, bitter and sour, and fatty acids^33^, and impaired olfactory performance have been observed in people with obesity^34–36^, or increased BMI^37–42^. However, other reports have produced conflicting evidence^43–47^. These participants also showed lower levels of well-being according to what has been already reported in other studies^48^.

In our approach, self-rated data were used as predictive variables even though they, are highly subjective evaluations that may produce large errors of measurement. Notwithstanding this limitation, self-rated evaluations, as well as all psychophysical testing procedures, are the only procedures in use in human studies since they are of simple application to large populations at a low cost and without causing inconvenience. In addition, the strong correlation that we found between self-rated oral sensitivity data and those obtained by the complete procedure of the “12-taste strip test” in the two groups is indicative of the fact that self-rated data can effectively be used. Moreover, the training and performance of our approach were tested by the metrics of evaluation, SMAPE, and MSE, which allowed us to verify, in both SL experiments, that the predicted values by the CatBoost regressor algorithm are strictly close to experimental values, the error percentages were 5.39%, and 27.81% and the average squared differences were 0.025 and 0.034 in control individuals and patients with chemosensory loss, respectively. These results indicated that the CatBoost regressor could efficiently learn from this kind of data and return a high-precision prediction of the target in the two groups even if they had a low size (mostly the group of patients) and were unbalanced in number and gender. However, Machine Learning techniques are known to require large datasets to fit the algorithms, and bias is expected to be larger for smaller datasets. Therefore, it would be beneficial to confirm our findings in larger samples.

The Permutation explainer method allowed us to achieve the importance and contribution of features in the learning of the CatBoost regressor model to predict the self-rated oral sensitivity in the Local Interpretability which regards a single instance and the Global Interpretability which regards the whole dataset. For the Local Interpretability, we have chosen the two more representative instances of the two groups, i.e. those in which the predicted values were more strictly close to experimental values. In the instance of the control group, the experimental value was 5 and that predicted 4.999, in the instance of the patient group the experimental value was 2 and that predicted 1.938. The smaller distance between the two values in the control individual could depend on the fact that in the control group, the CatBoost regressor learned better thanks to the larger number of observations. However, the metrics of evaluation of the training and performance of the algorithm computed in the patient group indicated that the algorithm predicted values of the self-rated oral sensitivity with high precision also in this group, and the evidence is that the predicted values of patients are very close to the experimental ones as shown by the value of MSE (0.034) in this dataset. The local interpretability of the permutation explainer allowed us to identify in the control individual, the self-rated sensitivity for astringency, the self-rated sensitivity for spiciness, the self-rated taste sensitivity, and the self-rated smell sensitivity as the four features most important to predict the value of the self-rated oral sensitivity, positively contributing with respect to the average of the predicted values of the whole dataset. Differently, in the patient instance, the self-rated sensitivity for astringency and the self-rated sensitivity for spiciness, and PTC ratings, which were the three most important features, provided a negative contribution to the predicted value of the self-rated oral sensitivity of this patient. The successive features in order of importance were different in the two instances: depression, the correct identification of high concentration of salt, age, the correct identification of low concentration of sweet, and the correct identification of high concentration of astringent stimulus in the control individual and the self-rated taste sensitivity, BMI, the correct identification of high concentration of capsaicin, age, the correct identification of high concentration of sour, and sex in the instance of the patient group.

In the Global Interpretability, the Permutation Explainer allowed us to identify the average of the importance and contribution of features in the learning of the CatBoost regressor model to predict the values of the self-rated oral sensitivity in the whole dataset of the control group and in the whole dataset of the patient group. It is interesting to note that only the self-rated sensitivity for astringency, the self-rated sensitivity for spiciness, and the self-rated taste sensitivity shared more than 75% of explanatory power with each other in both groups (83.33% in control individuals and 77.94% in patients). However, in control individuals, the self-rated sensitivity for astringency and the self-rated sensitivity for spiciness had a similar impact on the model output (32.22% and 31.11% were their explanatory power), and the self-rated taste sensitivity had a lower impact (20% was the explanation power). Differently, in patients, the self-rated sensitivity for astringency was the most important feature with an explanatory power of 39.7%, while the self-rated sensitivity for spiciness had a lower impact, with 27.94% explanatory power. The self-rated taste sensitivity was the third significant feature with a 10.29% explanation power. The self-rated smell sensitivity was the fourth feature in both groups, but it had an extremely low explanatory power (about 3% in both groups). Also, the Global Interpretability highlighted that the other features that significantly impacted the model output were different in the two groups, though they had a very low explanation power. They were the correct identification of the high concentration of astringent stimulus, depression, the correct identification of low concentration of sweet, age, the correct identification of high concentration of sweet in the control group, and PTC ratings, age, BMI, the correct identification of high concentration of capsaicin and sex in the patient group.

It is noteworthy that the average level of well-being was a significant feature in predicting the self-rated oral sensitivity in control individuals who had higher levels, while the BMI was significant in the patients, who showed higher values.

In conclusion, our results indicated that the CatBoost regressor is a reliable strategy to analyze with high precision the subjective ratings of oral sensitivity in healthy individuals and patients with chemosensory loss. Furthermore, the proposed approach, which provides real-time decision-making, allowed us to deeply understand the significance and contribution of the single components of gustatory, olfactory, and trigeminal of the oral sensitivity, also highlighting the differences between the two groups. The self-rated trigeminal component that we determined as evaluation for astringency and spiciness stimuli, was the most significant in the two groups, contributing more than 60% to the subjective ratings of oral sensitivity. However, astringency and spiciness gave a similar contribution in the healthy participants, while astringency was most important in patients, compared to spiciness. The identification of the contribution to the oral sensitivity of the single components, identifying the most important, offers the great advantage to permit, in future studies, the reduction of the experimentation times in each participant, from 20 min, required by the complete procedure of the “12-taste strip test” to 3 min required by a single stimulus. The self-rated taste component was more important in healthy participants, while in both groups smell provided the least important part of oral sensitivity. It is extremely interesting that astringency and spiciness are such good discriminators. This needs to be explored more deeply and will be the topic of future research projects. Since impairment in oral perception can affect the quality of life and eating behavior and be a risk factor for reducing well-being^10,11^, the identification of the relationships among, and the importance of, chemosensory and oral trigeminal components of oral perception provide important data to be applied in the clinical practice. However, a larger investigation is required to corroborate the results.

It is common knowledge that healthy individuals differ greatly in their physiological oral sensitivity. On the other hand, a loss of taste and/or smell has been connected to several diseases or pharmaceutical remedies^4,5,7–9^. Solid psycho-physical procedures are key tools for conducting routine clinical examinations. These are lengthy procedures, though, and they need a big commitment from both patients and medical staff. We developed an SL regression model that allows us to accurately analyze the subjective ratings of oral sensitivity in healthy individuals and patients with chemosensory, also understanding the contribution of the single sensorial components. The obtained results are of great interest to the health system because they allow the use of specific stimuli, simplifying tests, especially during routine clinical measurements (e.g., annual exams or follow-up visits) thus reducing the commitment in terms of time and costs.

## Data Availability

The datasets generated and/or analyzed during the current study are available from the corresponding author on reasonable request.
